# Milk Transmission of HTLV-1 and the Need for Innovative Prevention Strategies

**DOI:** 10.3389/fmed.2022.867147

**Published:** 2022-03-11

**Authors:** Sebastian Millen, Andrea K. Thoma-Kress

**Affiliations:** FAU Junior Research Group “Retroviral Pathogenesis” and BMBF Junior Research Group in Infection Research “Milk Transmission of Viruses”, Institute of Clinical and Molecular Virology, Friedrich-Alexander-Universität Erlangen-Nürnberg (FAU), Erlangen, Germany

**Keywords:** HTLV-1, virus transmission, breastfeeding, breast milk, tonsils, small intestine, oral route

## Abstract

Breastfeeding is recommended by the World Health Organization for at least 6 months up to 2 years of age, and breast milk protects against several diseases and infections. Intriguingly, few viruses are transmitted *via* breastfeeding including Human T-cell leukemia virus Type 1 (HTLV-1). HTLV-1 is a highly oncogenic yet neglected retrovirus, which primarily infects CD4^+^ T-cells *in vivo* and causes incurable diseases like HTLV-1-associated inflammatory conditions or Adult T-cell leukemia/lymphoma (ATLL) after lifelong viral persistence. Worldwide, at least 5–10 million people are HTLV-1-infected and most of them are unaware of their infection posing the risk of silent transmissions. HTLV-1 is transmitted *via* cell-containing body fluids such as blood products, semen, and breast milk, which constitutes the major route of mother-to-child transmission (MTCT). Risk of transmission increases with the duration of breastfeeding, however, abstinence from breastfeeding as it is recommended in some endemic countries is not an option in resource-limited settings or underrepresented areas and populations. Despite significant progress in understanding details of HTLV-1 cell-to-cell transmission, it is still not fully understood, which cells in which organs get infected *via* the oral route, how these cells get infected, how breast milk affects this route of infection and how to inhibit oral transmission despite breastfeeding, which is an urgent need especially in underrepresented areas of the world. Here, we review these questions and provide an outlook how future research could help to uncover prevention strategies that might ultimately allow infants to benefit from breastfeeding while reducing the risk of HTLV-1 transmission.

## Introduction

Human T-cell leukemia virus type 1 (HTLV-1) is a neglected highly oncogenic retrovirus infecting at least 5–10 million people worldwide, which is probably an underestimation (1). Endemic regions are located in Japan, Melanesia, Central Australia, Sub-Saharan Africa, parts of South America (e.g., Brazil), the Caribbean, and the Middle East (2). A high frequency of people infected with HTLV-1 (PHTLV) worldwide lives in resource-limited settings or belongs to social or ethnic minorities (2, 3). Problematically, many of the PHTLV are unaware of their infection since HTLV-1 is not screened routinely in every endemic country (1). Therefore, PHTLV might pass the virus to other vulnerable groups *via* blood products, sexual transmission and mother-to-child transmission (MTCT). Upon infection, HTLV-1 integrates into the host cell genome and PHTLV are life-long suffering from the burden of HTLV-1-infection and an increasingly recognized impairment of quality of life (4). Carriers face the risk of developing diseases with high morbidity and mortality, especially if infection has been acquired during infancy and due to the high incidence of co-infections (5). Approximately 10% of PHTLV develop incurable diseases including HTLV-1-associated inflammatory conditions like HTLV-1-associated myelopathy/tropical spastic paraparesis (HAM/TSP), or the fatal neoplasia Adult T-cell leukemia/lymphoma (ATLL). Infection upon MTCT poses an exceptional high risk for the infants to develop ATLL during their lives (6). In endemic areas of HTLV-1-infection, such as southwestern Japan, MTCT has been demonstrated to be the primary mode of transmission (7), while the main route of transmission for other countries like Brazil (ca. 0.8–2.5 million PHTLV) or Central Australia is still a matter of discussion (3, 8, 9). Although intra-uterine transmission of HTLV-1 has been described and perinatal transmission cannot be fully excluded (10, 11), the majority of HTLV-1 MTCT occurs *via* breastfeeding since the level of infection among babies that are exclusively formula fed is low (6, 7). In breastfed infants, MTCT occurs at rates varying from 7.4 to 32%, compared with a rate of less than 2.5–5% among bottle-fed children (6, 8, 12). Risk factors for HTLV-1 transmission *via* breastfeeding are (1) high proviral loads (PVL) in milk and blood, (2) low income, (3) breastfeeding over a longer period, (4) previous HTLV-1-infected offspring, (5) HLA-concordance between mother and child, (6) coinfection with the nematode Strongyloides sp., or (7) being a HAM/TSP patient (6, 13). Infection most likely results from the prolonged exposure of infants to HTLV-1 infected cells in breast milk after the loss of protective maternal antibodies (9, 14).

**FIGURE 1:**
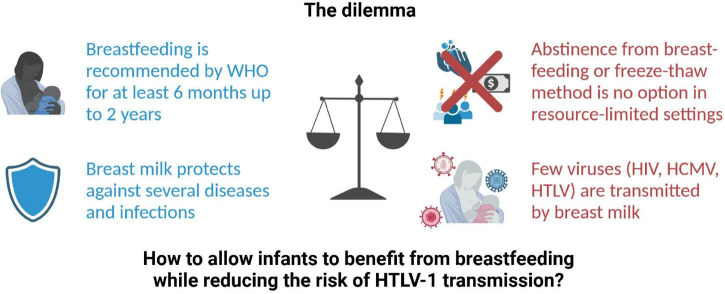
The dilemma of breastfeeding and HTLV-1 transmission. HCMV, Human Cytomegalovirus; HIV, Human Immunodeficiency virus; HTLV, Human T-cell leukemia virus; WHO, World Health Organization. Created with BioRender.

Overall, there is a dilemma whether the benefits from breastfeeding outweigh the risk of virus transmission (Figure 1). On the one hand, breastfeeding is recommended by WHO for the first 6 months up to 2 years of age since breast milk provides optimal nutrition to the infant and protects against severe diseases and infections, especially diarrheal infections attributed to contaminated drinking water (15). On the other hand, few viruses are transmitted *via* breast milk including Human Cytomegalovirus (CMV), Human Immunodeficiency Virus (HIV), HTLV-1, and the related HTLV-2 (16, 17). For arboviruses like Zika Virus, Dengue Virus, or Yellow Fever Virus, transmission *via* breast milk is under debate (18). However, abstinence from breastfeeding is no option in resource-limited settings due to impaired access to clean drinking water. In addition, social stigmatization of non-breastfeeding mothers, in certain cases, might serve as a hurdle to cease from breastfeeding, or impact adherence to the recommendation of avoidance of breastfeeding. Moreover, freezing of milk from HTLV-1-infected mothers has been shown to reduce risk of transmission in Japan (19), but this may not be feasible in developing countries. Therefore, this mini-review provides an overview of the oral route of HTLV-1 transmission *via* breastfeeding and an outlook how future research could help to uncover prevention strategies of MTCT, which proves to be one of WHO main priorities (12). These implementations should ultimately allow infants to benefit from breastfeeding while reducing the risk of HTLV-1 transmission.

## Which Cells in Which Organs Get Infected *via* the Oral Route of HTLV-1 Transmission?

**FIGURE 2:**
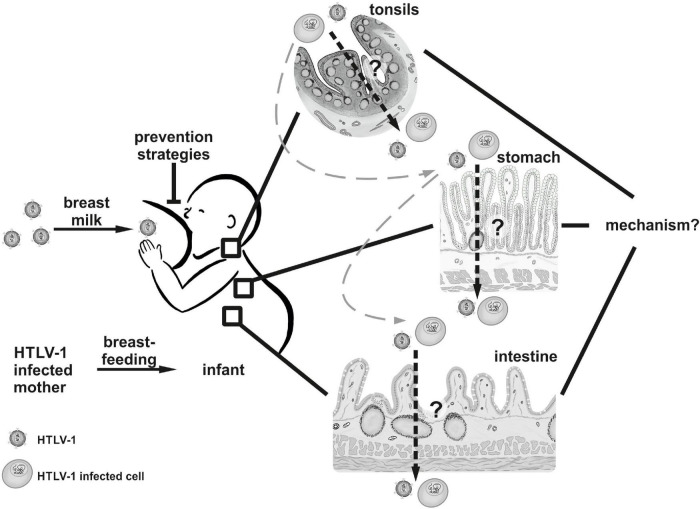
The oral route of HTLV-1 transmission. HTLV-1 is transmitted from mother to child *via* breastfeeding. It remains to be determined (1) whether HTLV-1 or HTLV-1-infected cells cross epithelial barriers in the tonsils, the stomach, or the intestine of the infant *in vivo*, and (2) by which mechanisms the virus is transmitted *in vivo*. *Tissues and cells displayed in this figure were drawn manually by Annika P. Schnell with Procreate*^®^ *Version 5.2.4 (Hobart, Tasmania) on the Apple iPad Pro 11” 2020 (Cupertino, United States).*

Ingestion of breast milk constitutes the major pathway of HTLV-1 MTCT, and common marmosets, rabbits and rats can be experimentally infected with HTLV-1 *via* the oral route (20–23). Upon ingestion, breast milk first comes to pass the oral cavity of the infant. Due to their location, architecture and enrichment in lymphocytes, palatine tonsils represent the first possible viral entry site (Figure 2). Indeed, tonsils seem to constitute an HTLV-1 reservoir as proviral DNA could be detected in extrafollicular areas in tonsil sections from HTLV-1 seropositive patients (24). Several lines of evidence additionally suggest that labial salivary glands (LSG) represent a non-lymphoid viral reservoir since proviral DNA as well as HTLV-1 gene products have been detected in LSG tissues and acini cells of HAM/TSP patients and individuals presenting with Sjögren’s syndrome (25, 26). Moreover, in co-culture with the chronically infected T-cell line HCT-5, HTLV-1 Gag protein was rapidly transferred to salivary gland epithelial cells, indicating that this cell type might—at least *in vitro*—be susceptible to HTLV-1 infection (27). Yet, it remains to be experimentally determined whether these tissues are permissive for primary HTLV-1 infection, especially in the context of breast milk carrying infected cells.

Having passed the suckling’s pharynx, subsequent potential HTLV-1 entry sites are located in the gut and small intestine (Figure 2). To this end, infected cells or infectious virus have to withstand the passage through the infant’s stomach. This idea is bolstered by experimental data indicating that medium pH = 3 had little influence on leukocyte survival as well as on HTLV-1 transmissibility from *in vitro* transformed breast milk macrophages to stimulated PBMC (28, 29). In fact, several animal models including rat, sheep and baboon, showed that colostral cells and milk leukocytes that were delivered to the newborn by lactation or oral application were able to pass the gastric epithelium and reach the neonate’s tissues and circulation (30–32).

After gastric transit, infected cells or infectious virus encounter the small intestine. Here, the gut-associated lymphoid tissue (GALT), a major secondary lymphatic organ, arises as an ideal candidate for potential HTLV-1 entry. Beneath the protective mucus film containing a multitude of antimicrobial peptides like defensins and lysozymes, the intestinal epithelial cell layer followed by the lamina propria is located (33). These structures contain many innate immune cells including macrophages and dendritic cells as well as intestinal intraepithelial lymphocytes (IEL) (34). Since most IELs at the surface mucosal compartment are partially activated T-cells it is conceivable that these cells are easily susceptible to HTLV-I infection (29). Importantly, the GALT contains accumulated lymphoid follicles clustering as Peyer’s patches (PP) in the ileum. Of note, in the murine system, maternal leukocytes that are transferred upon ingestion of breast milk preferentially localize to the offspring’s PPs (35). PPs are surrounded by the follicle-associated epithelium (FAE), which constitutes a junction between the GALT and the luminal microenvironment (36). The FAE is not only characterized by a large number of infiltrated B- and T-cells as well as macrophages and dendritic cells (DCs) but also encompasses specialized cells termed M-cells (microfold cells). Interestingly, M-cells were shown to serve as entry ports to the host for several viruses, including HIV-1, reo- and poliovirus in humans, as well as murine leukemia virus in mice (37–40). Therefore, M-cells might represent an attractive target also for HTLV-1 transmission, which remains to be addressed. General permissiveness of intestine tissues for HTLV-1 infection was demonstrated as the virus was detected in the mesenteric lymph nodes and the GALT after intravenous viral inoculation of squirrel monkeys or rabbits, respectively (41, 42).

## How Do Cells Get Infected With HTLV-1 *via* the Oral Route?

Establishing an HTLV-1 infection *via* the oral route could occur in several ways, which has been nicely summarized in recent reviews (43, 44). First, HTLV-1 infected cells could readily pass a physically damaged or disrupted epithelial cell layer. Of note, in humans, intestinal permeability is very high shortly after birth but drops soon during the infant’s development (45). Since subsequent gut closure is a very gradual process this might serve as an entry point for infected cells. In fact, an *in vitro* study conducted by Afonso and colleagues showed that infection of cerebral endothelial cells mimicking the blood-brain-barrier led to barrier breakdown (46). On the other hand, using intestinal epithelial cell lines, no loss of epithelial cell layer integrity was observed (47), indicating cell type specific outcomes in different tissues. Interestingly, a recent study suggested that co-infection with HIV-1 and HCMV evoked a disruption of the mucosal layer, allowing viral spread to the tonsils using an *in vitro* model (48). Similarly, a report by Ohata and colleagues analyzing an HTLV-1/HCMV co-infected patient described a gastroenterocolitis setting, indicating that viral interplay might affect mucosal integrity (49). Interestingly, mixed feeding (powdered infant formula and breastfeeding) is associated with higher rates of HIV-1 transmission (50). This finding is probably due to altered gut mucosal lining and permeability as a result of an increased gut pH and altered microbiota, which has been observed in infants fed with formula milk or prematurely with solid foods (51–53). Therefore, it would be interesting to determine whether mixed feeding also impacts transmission of HTLV-1.

Apart from that, HTLV-1 infected macrophages, although not formally demonstrated yet, but which might be present in breast milk, could transmigrate through the intact epithelium. This mode of action was observed to be employed by HIV-1 using *ex vivo* organ tissue models, however, until now there is no experimental data in regard to HTLV-1 (54). Another plausible mechanism establishing HTLV-1 infection is productive infection of the epithelial cell layer. Indeed, an *in vitro* study observed HTLV-1 infection of enterocytes with newly generated virus being released from the basal surface (55). In contrast, Martin-Latil and colleagues could not observe productive infection in their epithelial cell line models but rather demonstrated that HTLV-1 crosses the epithelial barrier by transcytosis (47). Such transit of virions incorporated into vesicles from the apical-to-basal surface has been delineated also for HIV-1 in a monostratified epithelium *in vitro* model (56).

Irrespective of the mechanism, HTLV-1 is dependent on close cell-cell contacts to establish infection. A well-described specialized structure, which is formed after contact between an HTLV-1 infected and a target T-cell, is the virological synapse (VS) (57, 58). Adding to the model of virus transmission by close cell-cell contacts, *in vitro* data put forward the idea that clusters of virus containing components of the extracellular matrix (ECM) are transferred to target cells, so called viral biofilms (VB) (27, 59–61). However, it remains to be determined whether formation of the VS or of infectious VB occurs during the oral infection route. Moreover, it is unclear whether extracellular vesicles secreted from HTLV-1 infected cells, which enhance viral spread *in vitro*, also modulate primary infection *via* the oral route (62). Of special note, it was shown that subepithelial DCs were infected in a co-culture model after transcytosis of HTLV-1 through an intestinal epithelial cell layer (47). Plasmacytoid and myeloid DCs from the peripheral blood of HTLV-1 seropositive individuals can be infected by HTLV-1 and are able to transmit the virus to and productively infect CD4^+^ target T-cells (63, 64). This mode of action was demonstrated to occur not only after *cis* infection of DCs but also as an *in trans* model with HTLV-1 virions binding to DCs followed by transfer of the virus to target T-cells prior to infection of the DCs themselves (64, 65). Moreover, plasmacytoid DCs from uninfected people are susceptible to infection, can be experimentally infected with viral biofilm and pass the infection to T lymphocytes *in vitro* (60). These findings altogether propose DCs to be key player in primary infection of HTLV-1 permissive tissues as well as in activation of innate immunity (66). Still, the role of DCs in the context of HTLV-1 transmission by breast milk remains elusive and needs to be experimentally investigated.

## Which Cells in Breast Milk Carry HTLV-1 and How Does Breast Milk Impact on Virus Transmission?

Breast milk constitutes of water, probiotic bacteria, breast milk cells, and macro- and micronutrients including lipids, fats, proteins and carbohydrates. The repertoire of breast milk cells is highly heterogeneous and contains (1) breast-derived cells including luminal epithelial cells (LEC; ductal non-secretory epithelial cells and lactocytes), myoepithelial cells from the ducts, squamous epithelial cells (from skin and nipple of the breast), progenitor and stem cells, and (2) blood-derived cells including immune cells (e.g., macrophages, lymphocytes, and neutrophils), and hematopoietic progenitor and stem cells (67–69). Moreover, breast milk is also rich in biologically active components like growth hormones, immunoglobulins, microbiota, and human milk oligosaccharides (HMOs), the latter representing important nutrients in human milk, which also function in direct pathogen binding and as prebiotics during the establishment of the infant’s microbiome. Interestingly, HMOs have also antiviral properties, e.g., against HIV, rotavirus, norovirus, influenza, and respiratory syncytial virus (68, 70, 71).

In breast milk, cell free HTLV-1 virions have not been detected yet, but HTLV-1-infected mononuclear cells (72, 73). There are large numbers of macrophages present in early lactation that decrease with the maturation of the milk (68). In experimentally infected breast milk macrophages (BMM), integrated provirus as well as Gag p24 (intracellular) and Gag p19 (secreted) were detectable. Immortalized BMM retained their phagocytic activity and were able to transmit HTLV-1 to T-cells (29). Next to lymphocytes and macrophages of human breast milk, also LEC can be experimentally infected with HTLV-1 and may pass the infection to other cells (43, 74). Interestingly, productively HTLV-1-infected LEC could be kept in continuous culture and synthesized an extensive extracellular matrix containing Collagen IV, which is also part of the viral biofilm (59, 61, 75). LEC were able to infect milk and intestinal epithelial cells as well as blood and milk leukocytes, and HTLV-1-infected LEC have been detected in breast biopsies of an ATLL patient with gynecomastia. Therefore, it is possible that LEC could play a role as an HTLV-1 reservoir *in vivo* (75, 76). Yet, it remains to be determined whether also breast milk lymphocytes are infected with HTLV-1. Recent studies from mothers vaccinated against SARS-CoV-2 revealed that breastmilk T-cells (BMTC) contain higher concentrations of antigen-experienced effector and central memory T-cells (77). This is in agreement with an earlier study in people with HIV infection, and it may be speculated that BMTC arise from a tissue-resident population in breast tissue rather than from the peripheral blood. Thus, breast biopsies of HTLV-1-infected mothers as well as a detailed characterization of the HTLV-1-infected cells in breast milk would broaden our understanding of the initial steps of MTCT.

Next to breast milk cells, other milk components may impact HTLV-1-transmission. Testing of paired blood and milk samples from HTLV-1-infected mothers using a newly developed anti-HTLV IgG capture assay showed the presence of anti-HTLV-1/2 IgG in milk in the same proportion as blood but in lower quantity and that PVL in milk correlates with blood (14). Anti-HTLV-antibodies may protect transmission *in utero*, but decline after birth, and levels are low in breast milk. Therefore, prolonged breastfeeding and decline of protective antibodies may finally increase the risk of HTLV-1-transmission over time (14). Several milk components have been shown to interfere with HTLV-1 transmission, amongst them the soluble milk protein lactoferrin, transforming growth factor beta (TGF-β), and prostaglandin E2 (PGE2), which enhance HTLV-1 transmission to cord blood lymphocytes and transactivate the HTLV-1 long terminal repeat promotor (78–80). PGE2 has also been shown to enhance Gag p19 secretion, depending on viral replication (80). Interestingly, *lactoferrin* gene expression can also be induced by the HTLV-1 Tax transactivator, even when Tax is extracellularly administered, suggesting the existence of a paracrine loop in the lactic compartment between infected cells and lactoferrin-expressing cells of the mammary epithelium (81). Analysis with recombinant vaccinia viruses expressing HTLV-1 Env suggested that lactoferrin diminishes fusogenic activity of Env, but this has not been verified with HTLV-1-infected cells (78). Yet, a systematic evaluation of pro-and antiviral factors in breast milk and their impact on HTLV-1 MTCT is lacking.

## How to Inhibit Oral HTLV-1 Transmission Despite Breastfeeding? Past, Present, Future

A prerequisite for installing prevention strategies of HTLV-1 MTCT is to raise public awareness about virus transmission in endemic regions and, especially, to gain more knowledge about HTLV-1 prevalence in pregnant women worldwide. Still, there is a lack of cost-effective implementation strategies targeting HTLV-1 transmission in public health programs of many different realities around the world. An important step would be antenatal screening programs of pregnant women and education or counseling campaigns of seropositive mothers about the potential risks of viral transmission by breastfeeding, which has only been implemented for few endemic regions of HTLV-1-infection yet (6, 8). In fact, several studies showed that antenatal screening programs would not only prevent MTCT events but would even be financially beneficial to national health authorities (82, 83).

Nationwide antenatal screening programs have been installed in Japan in 2010 (84, 85), while in France, Brazil and Chile, people with certain risk factors or from endemic regions are screened (86). In addition, screening of breast milk donations is recommended in the United Kingdom and in France, at least for donors from endemic regions (12). Routine screening of milk banks originating from donors of endemic regions should be advisable. Brazil has been recommending HTLV-1/2 infected mothers to avoid breastfeeding in 2019 (3), a strategy that proved successful in Japan earlier: Abstinence from breastfeeding by PHTLV mothers reduced the prevalence of HTLV carriers from 20–25% to 4% in the Nagasaki population in Japan (8). In Japan, HTLV-1 infected mothers are advised not to breastfeed or to do so only for less than 3 months (7). These recommendations are based on studies showing that avoidance of breastfeeding and exclusive formula feeding, short-term breastfeeding up to 3–6 months, or, in few studies, the freeze-thaw method reduce MTCT (19, 87, 88).

However, these practices may not be feasible in any setting: avoidance of breastfeeding may not only be harmful to the infant due to the loss of maternal passive immunity but also due to the risk of infection-related infant mortality, e.g., as a result of limited access to clean water, or limited financial resources (89, 90). Several studies have described severe diarrheas and weight loss upon weaning (91). Next to economic problems, social stigmatization in some cultures when mothers do not breastfeed their children should not be ignored (92). On the other hand, seropositive mothers, who are aware of their infection, might be hampered to breastfeed due to their fear of transmitting the virus to their offspring or even feel guilty upon inadvertent transmission due to the lack of efficient education and MTCT prevention strategies. Taking into account the different realities around the world, it should be acknowledged that every recommendation favoring or possible obstacle precluding breastfeeding should be carefully weighed against each other. It is important to asses a seropositive mother’s particular risk of MTCT considering individual settings. Ideally, future prevention strategies should focus on inhibition of HTLV-1 MTCT despite breastfeeding to allow optimal nutrition of the infants.

But how could this be achieved? Prevention strategies could target either the infected mother or the newly infected infant. For HIV, WHO guidelines on HIV and Infant Feeding recommend “Mothers living with HIV should breastfeed for at least 12 months and may continue breastfeeding for up to 24 months or longer while being fully supported for antiretroviral therapy (ART) adherence” (93). In fact, this contrasts with recommendations provided in several high-income countries, including the United States, which advise complete cessation from breastfeeding to mothers living with HIV regardless of viral load and ART therapy (94, 95). However, unlike HIV, HTLV-1 predominantly expands by mitotic division of the infected cells rather than by new infections, thus, it remains to be determined whether ART could interfere with HTLV MTCT. Currently, clinical studies addressing the impact of ART on the PVL in breast milk of HTLV-1-infected mothers or, vice versa, assessing the use of prophylactic treatment of the suckling, using for example integrase inhibitors, are lacking but might serve as a promising and cost-effective tool to prevent MTCT. Future challenging strategies to get rid of the integrated virus within infected mothers could include excision strategies using HTLV-1-specific recombinases that have already been evolved for HIV (96, 97), zinc finger nucleases (98) or genetic editing strategies using CRISPR/Cas 9 (99). Hence, it may be easier to prevent *de novo* infection of the infant. From a practical point of view, mothers being aware of their infection should be equipped with easy-to-use, cheap, heat-resistant prevention tools, which can be applied while breastfeeding. These prevention tools do not exist yet and have to be developed. A specific tool to block HTLV-1 MTCT could be based on the anti-gp46 (env) neutralizing monoclonal antibody (mAb) termed LAT-27, which prevents *in vivo* transmission of HTLV-1 in a simple humanized mouse model of HTLV-1 infection (100). Of note, LAT-27 was not able to prevent MTCT in an orally HTLV-1 infected rat model when injected intraperitoneally, indicating that the mode of application might be decisive to inhibit MTCT (101). It remains to be determined whether MTCT could be prevented by neutralizing HTLV-1 specific antibodies in humans, which is an effective strategy to inhibit Hepatitis B Virus (HBV) transmission using Hepatitis B immune globulin (HBIG) (102). Moreover, it may also be interesting to see whether these antibodies can be applied as a spray into the oral cavity while suckling. Moreover, little is known about therapeutics that prevent entry of the infected cell, e.g., by interfering with the HTLV-1-receptor, the virus, or the infected cells, and which could ideally be applied topically on the breast of the infected mother or within the oral cavity of the suckling infant.

## Concluding Remarks

Overall, there is a great need to develop novel prevention strategies of HTLV MTCT with the potential to protect the most vulnerable members of our society, newborn infants, from live-long, live-threatening, and incurable infections.

## Author Contributions

SM and AT-K wrote the article. Both authors contributed to the article and approved the submitted version.

## Conflict of Interest

The authors declare that the research was conducted in the absence of any commercial or financial relationships that could be construed as a potential conflict of interest.

## Publisher’s Note

All claims expressed in this article are solely those of the authors and do not necessarily represent those of their affiliated organizations, or those of the publisher, the editors and the reviewers. Any product that may be evaluated in this article, or claim that may be made by its manufacturer, is not guaranteed or endorsed by the publisher.
